# Lessons Learned—The Impact of the Third Wave of the COVID-19 Pandemic on German Waldorf Parents’ Support Needs and Their Rating of Children’s Health-Related Quality of Life: A Cross-Sectional Online Survey

**DOI:** 10.3390/ijerph20064756

**Published:** 2023-03-08

**Authors:** Jan Vagedes, Karin Michael, Mohsen Sobh, Mohammad O. A. Islam, Silja Kuderer, Christian Jeske, Anne Kaman, David Martin, Katrin Vagedes, Michael Erhart, Ulrike Ravens-Sieberer, Tomáš Zdražil

**Affiliations:** 1 Academic Research in Complementary and Integrative Medicine (ARCIM Institute), 70794 Filderstadt, Germany; 2 Department of Neonatology, University Hospital Tübingen, 72076 Tübingen, Germany; 3 Community Hospital Witten-Herdecke, 58313 Herdecke, Germany; 4 Department of Health and Pedagogy, Freie Hochschule Stuttgart, 70188 Stuttgart, Germany; 5 Department of Child and Adolescent Psychiatry, Psychotherapy, and Psychosomatics, University Medical Center Hamburg-Eppendorf, 20246 Hamburg, Germany; 6 Department of Human Medicine, Faculty of Health, Witten-Herdecke University, 58455 Witten, Germany; 7 Department of Pediatric Oncology, University Hospital Tübingen, 72076 Tübingen, Germany; 8 Department of Public Health, Alice Salomon University of Applied Sciences, 12627 Berlin, Germany; 9 Department of Psychology, Apollon University of Applied Sciences, 28359 Bremen, Germany

**Keywords:** COVID-19, pandemic, coronavirus, homeschooling, Waldorf school, child health-related quality of life, parent support need

## Abstract

Background: COVID-19-related lockdowns and homeschooling have imposed a substantial burden on school-aged children and parents. Waldorf education is a reform-educational concept. Little is known about the situation of German Waldorf families under pandemic conditions. Methods: A cross-sectional, online, parent-proxy survey was conducted regarding the third pandemic wave. The primary outcome was parents’ support needs, assessed with questions from the German COPSY (**CO**VID-19 and **PSY**chological Health) study; the secondary outcome was children’s HRQoL (KIDSCREEN-10, proxy version). Results: We analyzed questionnaires from 431 parents of 511 Waldorf students aged 7 to 17 years. While 70.8% of Waldorf parents (WPs) reported a general need for support in dealing with their children, 59.9% of COPSY parents (CPs) indicated this need. WPs’ support needs in dealing with their children’s academic demands were similar to CPs’ needs but relatively higher in terms of dealing with emotions and moods, behavior, and relationships within the family. WPs sought support mainly from school and teachers (65.6%). Support needs were high, although WPs rated their children’s HRQoL higher than CPs. Conclusions: Our results underline the substantial pandemic-related burden on families across school types. WPs participating in this survey gave evidence that supports should focus on academic demands as well as psychosocial issues.

## 1. Introduction

During the COVID-19 pandemic, particularly during lockdown and homeschooling, parents and children were exposed to significant additional demands that led to deteriorating mental health and increased support needs [1,2,3,4]. In addition to coping with social isolation and disruptions in family and work routines [5], parents had to focus on their children’s needs and demands, such as supporting and supervising them during homeschooling and managing behavioral problems [6,7]. These time-consuming tasks meant that parents had less free time and fewer hours of sleep [7,8]. Moreover, most parents did not have the skills to serve as substitute teachers in addition to their job and family responsibilities [9,10]. Therefore, parents were at an increased risk for stress and depression because the demands they had to meet exceeded their available resources [11].

Several studies have examined the impact of the pandemic and school closures on parents and their children, the difficulties faced by parents, and the support they needed [12,13,14,15,16]. An Australian study found an association between parents’ perceived support from the school during homeschooling and their level of psychological distress and wellbeing [17]. Some authors report positive experiences with school closures and remote schooling, which they see as a perspective for future-oriented modern schooling [18]. However, for the majority of families, the adverse effects of pandemic-related restrictions seem to be in the foreground. In a Europe-wide study, 6720 parents from seven European countries were predominantly found to experience increased psychological distress during homeschooling, have had little support from schools, and, overall, have had rather negative experiences with homeschooling [19].

For the children and adolescents, social distancing measures and homeschooling meant a major break in the sensitive phase of personality and identity development in the context of the social environment. This was not without consequences. In Germany, the COPSY (**CO**VID-19 and **PSY**chological Health) study is a population-based longitudinal study to examine the impact of the pandemic on health-related quality of life (HRQoL) and the mental health of children and adolescents aged 7 to 17 years. Self-report and parent-proxy surveys were conducted from 05–06/2020 [20], 12/2020–01/2021 [21] and 09–10/2021 [4] to capture the three waves of the pandemic so far. The study results indicate a substantial mental health deterioration in the children compared with pre-pandemic levels and considerable pandemic-related stress on the parents [4,20].

Thus, there is growing evidence of the burden experienced by parents and children during the pandemic. However, to our knowledge, only a few studies have examined the impact of the COVID-19 pandemic on the support needs of Waldorf parents in Germany.

Waldorf schools are based on the Waldorf education developed by Rudolf Steiner in Germany around 1920 and are classified as reform pedagogy. Waldorf education aims at a balanced integration of intellectual/cognitive, artistic/imaginative, and life skills/practical aspects based on Steiner’s reflections on the development of the child [22,23,24,25,26]. Especially with younger children, an emphasis is placed on teaching and learning based on manifold real-life sensory experiences. Only a limited use is made of computers in the classroom [27], which may have made homeschooling during the pandemic, with its need for an increased use of computers and other digital devices, particularly challenging for Waldorf students and their parents. Waldorf schools are non-governmental, private schools and depend on the support of the students’ parents, not only financially. To shed light on the situation of Waldorf school families in Germany during the pandemic, we conducted a cross-sectional online survey and collected data on parents’ support needs during the third wave of the pandemic. In addition, parents were asked how they rated their children´s HRQoL using the proxy version of the KIDSCREEN-10 index.

## 2. Methods

### 2.1. Study Design and Sample

The study was based on a cross-sectional online survey conducted by members of the University of Tuebingen, the University of Witten–Herdecke, the Freie Hochschule Stuttgart, and the ARCIM Institute (Academic Research in Complementary and Integrative Medicine), and it was approved by the ethics committee of Witten–Herdecke University (approval number: 78/2020). At the time of the survey in July/August 2021, the pandemic situation in Germany had eased somewhat, with low infection rates and rising vaccination rates. After several months of closure, schools gradually reopened, initially in different ways, e.g., by dividing students into two shifts with alternating classes. Some schools still remained closed and continued homeschooling. Parents of children between the ages of 7 and 17 attending a Waldorf school in Germany were eligible to participate in the study. Given the different start dates of the summer vacations in the German federal states, the specification of the state of residence was a further inclusion criterion. Data collection started on 14 July 2021. Questionnaires completed later than one day after the official start of the summer vacations in the respective federal states were excluded from the analysis. Participation in the survey was anonymous and voluntary. Neither the schools and their staff nor the participating families received any incentives or compensation. The link to the online questionnaire was distributed by e-mail in a “snowball system.” It was sent to the principals of the Waldorf schools and from them via the class teachers to the parents. At the beginning of the questionnaire, parents were asked to indicate how many children 7 to 17 years of age they have who attend a Waldorf school. If there was more than one, they were asked to fill out the questionnaire for each individual child.

### 2.2. Study Instruments

The survey was designed in German using LimeSurvey (version 5.1.2) (Limesurvey GmbH./LimeSurvey: An Open Source survey tool/LimeSurvey GmbH, Hamburg, Germany. URL http://www.limesurvey.org), an open-source online survey application that allows for development and conducting of online surveys and recording of their results in a database. The query period referred to lockdown/homeschooling during the third wave of the pandemic (November 2020 to June 2021). The questionnaire we used comprised different parts. It raised demographic information such as the children’s age, gender, BMI percentile, migration background, the parents’ age, marital status, education, and occupational status. In addition, self-developed items were used to collect data on living conditions, such as whether the family has access to a garden or terrace, how many rooms the house or apartment has, how many people live in the household, and whether the children have their own rooms. Other self-developed items were used to collect details about the type of schooling (classroom or home schooling) and about the implementation of homeschooling and digital distance learning (frequency, type and duration of digital distance learning offered by schools, number of children participating in digital lessons, extent of digital media use). Parents were asked about their self-perceived general support needs regarding their children and their family situation and, more specifically, about the areas in which they would like support and from whom. The questions on parents’ support needs were adopted from the COPSY study [20]. The children’s HRQoL was assessed using the validated KIDSCREEN-10 Index, which comprises 10 items from the domains of physical fitness, moods and emotions, social activities, emotions and interactions between children and their parents and peers, and school performance. Each item is rated on a five-point Likert-type response scale ranging from 1 (never; not at all) to 5 (always; extremely). A Rasch-scaled single score of overall HRQoL was generated, with a high score indicating better HRQoL [28,29,30]. We used the KIDSCREEN-10 parent-proxy version, which has a Cronbach’s alpha reliability of 0.78 and a test-retest intra class correlation coefficient (ICC) of 0.67 [29].

### 2.3. Outcome Parameters

Our primary outcome measure was the support needs of Waldorf parents and the areas in which they needed support compared to the third-wave COPSY sample.

Secondary outcome measures included HRQoL score, types of schooling, the frequency with which digital distance learning was offered, how children received homeschooling assignments, frequency of video lessons offered, average length of each video lesson, number of children participating, children’s media consumption, and factors related to parents’ support needs.

### 2.4. Statistical Analysis

Baseline demographics of the Waldorf sample were compared with the third-wave COPSY sample [4] using Cohen’s d effect sizes ($d=\frac{M_{1}-M_{2}}{{\mathrm{SD}}_{\mathrm{pooled}}}$) for metric-scaled characteristics (small effect: |d| = 0.2, medium effect: |d| = 0.5, large effect: |d| = 0.8 [31]) and Cramér’s V (CV, [32]) for categorial characteristics (small effect: CV = 0.1, medium effect: CV = 0.3, large effect: CV = 0.5 [33]). We calculated Cramér’s V and its 95% confidence intervals to compare Waldorf parents’ support needs and the areas of needed support with the third-wave COPSY sample (primary outcome measure). A Welch’s two-sample *t*-test was conducted to compare the T-values of the KIDSCREEN-10 global HRQoL score with the third-wave COPSY sample. To find out which factors had an impact on parents’ support needs, we conducted a median split for the four parameters (“access to a garden or terrace,” “number of people living in the household,” “number of rooms in the house or apartment,” and “number of children 7 to 17 years of age”) and used Fisher’s exact test to compare the percentage of parents above and below the median with regard to the five different areas of needed support (1. Dealing with the relationships within the family; 2. Dealing with the child’s emotions and moods; 3. Dealing with the child’s behavior; 4. Return of the child from isolation; 5. Dealing with the child’s academic demands [20]). In addition, we analyzed the percentage of answered questions for the four parameters above and below the median split, respectively. All analyses were conducted with R [34] and R Studio [35].

## 3. Results

### 3.1. Study Population

First, the link to the online survey was e-mailed to all Waldorf school principals in Germany (*n* = 254). Of the schools that received the e-mail before summer vacation (*n* = 159), 27 gave feedback that the link had been forwarded to the teachers (response rate = 17%). We did not receive information on how many teachers forwarded the invitation with the link to parents. A total of 537 parents participated in the survey between 14 July 2021 and 31 August 2021. Of these, 19.7% (*n* = 106) were excluded from analysis because they did not meet the inclusion criteria (children’s age > 17 years: *n* = 6; non-specified federal state: *n* = 36; and completing the questionnaire after the official start of summer vacation: *n* = 64). The final sample consisted of 431 parents from 10 different federal states (Baden–Württemberg: 53.8%; Bavaria: 17.9%; Hesse: 9.3%; Lower Saxony: 6.5%; Saxony: 6.0%; Thuringia: 2.1%; Saarland: 1.9%; Saxony–Anhalt: 1.6%; Rhineland Palatinate: 0.7%; and Bremen: 0.2%) who completed the questionnaire for a total of 511 children aged 7 to 17 years (parents completed the questionnaire for an average of 1.2 ± 0.5 children). Participation in the survey took place an average of 5.8 ± 3.8 days before the start of summer vacation. The mean age of the children was 11.7 ± 3.0 years, 50.3% were female, and most of them had no migration background (92.8%). Parents had a mean age of 46.3 ± 6.7 years, and the majority were married (75.6%), had a higher education (69.1%), and reported full-time employment (36.3%) (Table 1). Most of them lived in a house or apartment with a garden or terrace (87.2%; 11.6% were without a garden or terrace, while 1.2% gave no answer) with 5.2 ± 1.9 rooms and with 4.1 ± 1.1 people in the household. Most of the children had their own room (87.3%). The demographics of the Waldorf sample and the third-wave COPSY sample were similar (Table 1).

### 3.2. Support Needs of Parents

With respect to the primary outcome measures, more than two-thirds of Waldorf parents (70.8%) reported needing support in dealing with their children during the third wave of the COVID-19 pandemic. The need for support was higher in the Waldorf sample than in the third-wave COPSY sample (70.8% versus 59.9%, CV = 0.19 [0.14 to 0.24], Table 2).

**Figure 1:**
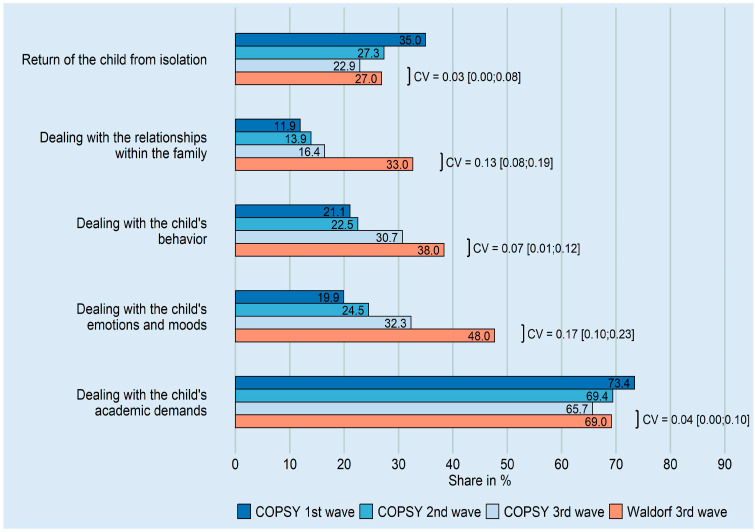
Support needs of Waldorf parents in dealing with their children during the third COVID-19 wave compared to the COPSY sample (first, second, and third wave) [4,20,21]. Multiple responses were allowed. Indicated is the share of parents who indicated that they always/rather often/occasionally needed support (Waldorf sample: *n* = 279). Cramér’s V (CV) and its confidence interval were used to compare the support needs of the Waldorf and COPSY samples during the third wave.

Among Waldorf parents who needed support, the most frequently indicated support-need issues were dealing with school demands, managing their child’s emotions and moods, and managing their child’s behavior (Figure 1). The need for support in dealing with relationships within the family was twice as high in the Waldorf sample as in the COPSY sample (33.0% vs. 16.4%, CV = 0.13). Waldorf parents also reported markedly higher support needs in dealing with the child’s emotions and moods (48.0% vs. 32.3%, CV = 0.17) and behavior (38.0% vs. 30.7%, CV = 0.07) (Figure 1).

Within the COPSY sample, the proportion of parents with support needs decreased from the first to the second or third wave (Table 2).

**Figure 2:**
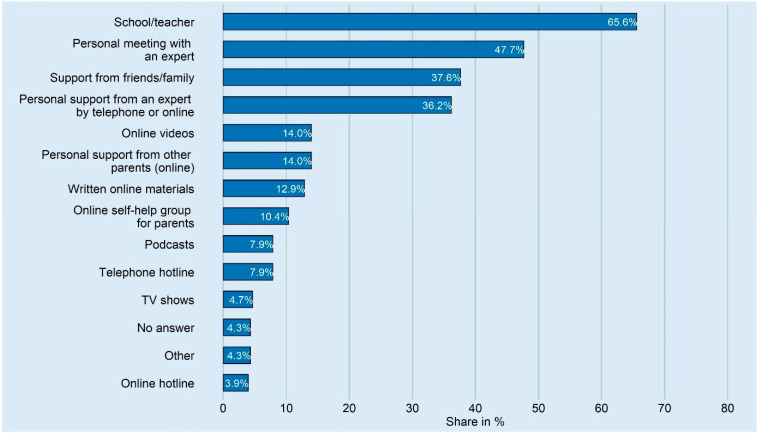
Information on how, or from whom, parents would like to receive support in dealing with their children during the COVID-19 pandemic. Indicated is the share of parents who answered that they always/rather often/occasionally needed support (*n* = 279).

When asked how or from whom they would like to receive support, Waldorf parents most often mentioned school and teachers (*n* = 183, 65.6%), face-to-face meetings with an expert (*n* = 133, 47.7%), and friends and family (*n* = 105, 37.6%) (Figure 2).

Subgroup analysis (Table 3) revealed that parents with larger households (four or more people) indicated marginally significantly higher support needs than parents with smaller households (less than four people) (*p* = 0.066, Cohens’ d = 0.27). Parents with larger households needed more support in managing the relationships within the family and in dealing with the child’s emotions, moods, and behavior. They reported less support needs in dealing with the return of the child from isolation and the child’s academic demands. In the larger households, the children were younger (age: $\bar{x}$ = 11.6; SD = 3.04) than in the smaller ones (age: $\bar{x}$ = 12.4; SD = 3.20). The difference in support needs was not significant between the parents with one school-aged child and those with more than one, between those with and without a garden or terrace, and between those with a large house (five or more rooms) and an average-sized house (less than five rooms).

### 3.3. HRQoL of Children

**Figure 3:**
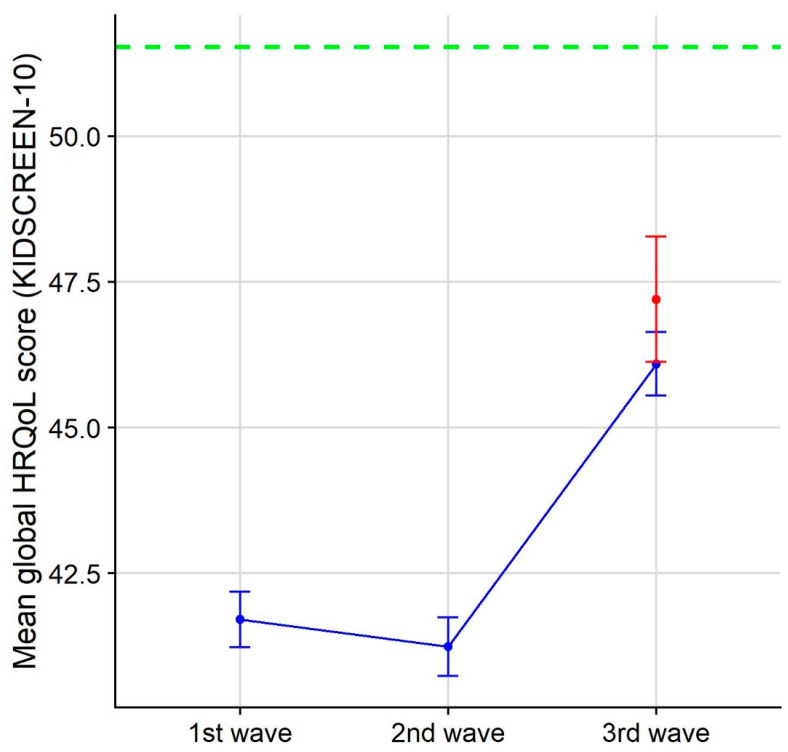
Mean global HRQoL scores (KIDSCREEN-10) and 95% confidence intervals for the Waldorf third-wave sample (in red) and for the COPSY first-, second-, and third-wave samples (in blue) (data provided by U. Ravens-Sieberer). The green-dashed line shows the German national norm data for the KIDSCREEN-10 HRQoL score of 8–18-year-old children from the proxy report (Table A7_B-32, [30]).

Results for the KIDSCREEN-10 Index were available for 413 children from 348 complete parent questionnaires (19.2% were discarded because of missing items). The mean global HRQoL score for children aged 7 to 17 was higher in the Waldorf sample than in the third-wave COPSY sample (Waldorf vs. COPSY, *t*(611) = 1.92, *p* = 0.05556, *d* = 0.11) (Figure 3, Table 4). However, despite an increase in HRQoL in the COPSY sample across the three waves, neither sample reached the value of the German national norm data for the KIDSCREEN-10 HRQoL score of 8–18-year-old children (Figure 3, [30]).

### 3.4. Teaching during the Third Wave

For the majority of children, classroom teaching was the predominant form of instruction during the four weeks preceding summer vacation (*n* = 458, 89.6%; homeschooling: *n* = 20, 3.9%; alternating lessons: *n* = 20, 3.9%; other forms: *n* = 8, 1.6%; no answer: *n* = 5, 1.0%). During the third pandemic wave, Waldorf schools offered digital distance learning for 35.2% (*n* = 180) of children on a daily basis, for 16.0% (*n* = 82) several times per week, and for 6.1% (*n* = 31) once per week. For 39.1% (*n* = 200) of children, no digital distance learning was provided at all (no answer: *n* = 18, 3.5%). Children received homeschooling assignments via e-mail (*n* = 317, 62.0%), digital platforms (*n* = 264, 51.7%), or hard copy (*n* = 188, 36.8%; other forms: *n* = 22, 4.3%; no answer: *n* = 6, 1.2%).

If digital distance learning was provided, telephone or video lessons were held on 3.7 ± 1.7 days per week, with 2.9 ± 1.3 different lessons per day and an average lesson length of 39.8 ± 18.2 min. The maximum number of children attending a lesson was an average of 13.1 ± 8.6. During homeschooling, children spent an average of 2.9 ± 1.8 h daily on schoolwork at home, including 1.7 ± 2.2 h in front of a computer/tablet/smartphone screen. This represents an increase in media use for more than half of the children (*n* = 269, 52.6%) compared to the pre-pandemic situation (unchanged media use: *n* = 187, 36.6%; less media use *n* = 42, 8.2%; no response: *n* = 13, 2.5%).

## 4. Discussion

This cross-sectional survey showed that more than two-thirds (70.8%) of a sample of Waldorf parents in Germany needed support in interacting with their children during the third wave of the COVID-19 pandemic. This need was indicated even though parents rated their children’s HRQoL as comparatively good, and despite the fact that the Waldorf sample reported good living conditions, with the majority of families living in a house with a garden or terrace and most children having their own room. Among parents in need of support, the most common request was for support in dealing with the child’s academic demands, followed by dealing with the child’s emotions and moods, behavior, relationships within the family, and return of the child from isolation.

Our findings are consistent with research worldwide that highlights parents’ concerns about their children’s academic performance and mental state and the need for support during homeschooling [19,37,38]. In Germany, the COVID-19 Snapshot Monitoring (COSMO) project was conducted; this included a repetitive cross-sectional survey with approximately 1000 respondents per survey wave. Particularly during the first wave of the pandemic, parents of school-aged children indicated a significantly higher burden compared to the general study population [39]. The Germany-wide COPSY study covered the first three waves of the pandemic. Parents participating in the three COPSY surveys reported a high need for support regarding their children’s academic demands. However, this specific need decreased over the course of the three pandemic waves (Figure 1), which could be related to a growing routine and confidence. In contrast, dealing with the child’s emotions and moods, behavior, and relationships within the family became more prominent across the three waves, with parents expressing an increasing need for support (Figure 1) (Ravens–Sieberer, personal communication). Other studies addressing the psychosocial situation within families during the pandemic reported similar findings [40,41,42]. Based on the German CoCo–Fakt Cohort study (Cologne–Corona Counselling and Support for Index and KontAKt Persons during the Quarantine Period), Nöthig et al. reported that family stress was significantly higher if at least one child was quarantined, emphasizing the additional burden of isolation. The authors also found a clear interaction between parental stress and children’s psychological distress [43].

The present study examines the pandemic-related situation of families with children at Waldorf schools, state-approved independent schools with a special educational concept. General and special characteristics of the German Waldorf parent population were collected in the WEiDE study (**W**aldorf-**E**ltern **i**n **DE**utschland, Waldorf parents in Germany), a survey conducted by Koolmann and colleagues in late 2014/early 2015 [44]. The results are based on 3685 questionnaires filled out by Waldorf parents. The authors found a lower proportion of parents with a migration background and a higher level of education among Waldorf parents compared to the national average. According to Koolmann et al., Waldorf parents do not have an above-average income. The decision for a Waldorf school was probably not made because a fee-paying private school was sought, but because parents were attracted by the comprehensive educational concept. The parents have high expectations of themselves and of the school, which, beyond being a place of pure knowledge transfer, should offer the children the best possible conditions for the development of their personality and individual strengths. Waldorf parents are value-oriented. They reflect on questions of meaning and attach importance to guiding children in school toward socially and ecologically responsible behavior and to discussing socially relevant topics with them, and they view their children’s Waldorf school with a correspondingly attentive eye [44]. According to the comparison of our demographic data with the WEiDE study, our sample can be placed in the context of German Waldorf parents (Table S1). Compared with the results of the COPSY study, parents in our sample issued similar concerns about their children’s academic demands and return from isolation but substantially higher support needs for psychosocial aspects, such as family relationships and dealing with the children’s emotions, moods, and behavior (Figure 1). These findings are in line with the characteristics reported by Koolmann et al. for German Waldorf parents who observe social interaction with great, perhaps over-reflective attention. Moreover, components of typical Waldorf teaching could hardly take place in homeschooling, especially the artistic/imaginative and life skills/practical components that are considered important in Waldorf education for the healthy development of children. Instead, digital learning and media use increased. This may have added to the parental concerns and self-perceived support needs. We conducted a subgroup analysis to look for other influencing factors. “Access to a garden or terrace,” “number of rooms in the house or apartment,” and “number of school-aged children (7–17 years)” did not contribute to the parents’ support needs, which is in line with the spacious living conditions reported by our sample. However, the number of people in the household did have an impact, with a higher number of people (four or more) resulting in a higher need for support. This is possibly due to the larger families having younger children. A relationship between the number and age of children, and the level of parental stress, has been described in recent studies stating that parents of younger or many children have higher levels of stress [20]. Finally, we cannot rule out a bias of predominantly highly reflective and sensitive parents with high self-perceived needs for support having participated in this study.

Interestingly, parents in our sample rated their children’s HRQoL as comparatively good, even though they experienced themselves as needing support. The fact that many children seem to have gotten through the pandemic-related restrictions relatively well may be related to the good living conditions and intensive efforts of their parents. However, given the high standards and self-expectations described above, these efforts may have led to exhaustion on the part of the parents resulting in an increased need for support.

This study was the first to examine the impact of the COVID-19 pandemic and homeschooling on Waldorf students in Germany and the support needs of their parents from the parents’ perspective. The comparison of our data with the representative WEiDE study showed that our sample fits into the overall context of German Waldorf parents, which supports the validity of our results. Our survey covered the third wave of the pandemic, so the results may provide an indication of the potential impact of longer-term restrictions. To allow for a comparison with the large-scale representative COPSY study, we used the same survey instruments.

Nonetheless, our results are subject to limitations. We cannot rule out the possibility of selection bias in the recruitment of our sample, as a snowball sampling procedure was used, and it is possible that mainly motivated school principals and teachers forwarded the e-mail to parents. In addition, our results are based on parents’ statements; a survey of children and adolescents was not included. More than two-thirds (71.2%) of the participating parents came from the two German states of Baden–Württemberg and Bavaria. Our results should, therefore, be viewed with caution in terms of generalizability to the whole of Germany. Though the main outcomes were assessed with validated measures, some information had to be gathered with ad-hoc-developed measures as there were no instruments available. The cross-sectional data analyzed precludes causal interpretation of the effects found.

## 5. Conclusions

Worldwide, parents of school-aged children reported an increased burden and a desire for support under the conditions of the COVID-19 pandemic. In Germany, Waldorf parents expressed a substantially higher need for support compared to the representative COPSY study population. They wanted this support primarily from the school and the teachers. A school with a special educational concept like the Waldorf school should expect to meet high expectations from parents and be prepared for an increased parental need for support in times like the pandemic with school closures and homeschooling. Waldorf parents in our study voiced relatively high support needs regarding psychosocial issues in the family during the third wave of the pandemic. Needs in this area also increased across the three waves in the COPSY study. The high burden on families under protective measures should, therefore, be an even greater focus of decision-makers in future pandemic policy decisions.

## Figures and Tables

**Table 1:** Demographics of the Waldorf and COPSY samples.

		COPSY Sample			Waldorf Sample	
		1st Wave (*n* = 1586) [20]	2nd Wave (*n* = 1923) [21]	3rd Wave (*n* = 2097) [4]	3rd Wave (*n* = 431 Parents, *n* = 511 Children)	3rd Wave: Waldorf vs. COPSY Sample
**Children**		*n* (%)/*M* ± *SD*				Cohen’s d/Cramér’s V
Age, in years ^a^	-	12.25 ± 3.30	12.67 ± 3.29	13.21 ± 3.30	11.66 ± 2.97	0.49
Gender	Male	791 (49.9)	964 (50.1)	1030 (49.1)	240 (47.0)	0.11
	Female	793 (50.0)	956 (49.7)	1055 (50.3)	257 (50.3)	
	Diverse	1 (0.1)	2 (0.1)	10 (0.5)	7 (1.4)	
	No answer	1 (0.1)	-	-	7 (1.4)	
BMI percentile ^b^	-	-	-	-	44.68 ± 30.01	-
Migration background	No	1332 (84.0)	1608 (83.6)	1741 (83.0)	474 (92.8)	0.19
	Yes	254 (16.0)	315 (16.4)	356 (17.0)	26 (5.1)	
	No answer	-	-	-	11 (2.2)	
**Parents**						
Age ^c^	-	43.99 ± 7.36	44.05 ± 7.35	44.41 ± 7.44	46.25 ± 6.68	0.26
Number of children ^d^	-	-	-	-	1.65 ± 0.78	-
Marital status	Single	140 (8.8)	-	-	24 (5.6)	-
	Married	1097 (69.2)	-	-	326 (75.6)	
	In a registered partnership	13 (0.8)	-	-	2 (0.5)	
	In a committed relationship	216 (13.6)	-	-	27 (6.3)	
	Divorced	108 (6.8)	-	-	40 (9.3)	
	Widowed	12 (0.8)	-	-	6 (1.4)	
	No answer	-	-	-	6 (1.4)	
Education ^e^	Higher education entrance qualification/Abitur	-	-	-	596 (69.1)	-
	Advanced technical college certificate	-	-	-	125 (14.5)	
	General certificate of secondary education	-	-	-	92 (10.9)	
	Certificate of secondary education	-	-	-	34 (3.9)	
	No school leaving certificate	-	-	-	4 (0.5)	
	No answer	-	-	-	9 (1.0)	
Occupation ^e^	Full-time employed	820 (51.7)	990 (51.5)	1069 (51.0)	313 (36.3)	0.33
	Part-time employed	453 (28.6)	555 (28.9)	616 (29.4)	234 (27.1)	
	Self-employed	67 (4.2)	78 (4.1)	81 (3.9)	185 (21.5)	
	Other employment	32 (2.0)	34 (1.8)	40 (1.9)	30 (3.5)	
	Housewife, househusband	109 (6.9)	130 (6.8)	142 (6.8)	41 (4.8)	
	On parental leave	29 (1.8)	26 (1.4)	31 (1.5)	16 (1.9)	
	Unemployed	42 (2.6)	68 (3.5)	66 (3.1)	12 (1.4)	
	Retiree/pensioner	34 (2.1)	42 (2.2)	52 (2.5)	10 (1.2)	
	No answer	-	-	-	21 (2.4)	

Missing values in the Waldorf sample: ^a^ *n* = 20 (3.9%), ^b^ *n* = 61 (11.9%), ^c^ *n* = 20 (4.6%), ^d^ *n* = 1 (0.2%); ^e^ Fathers and mothers of the Waldorf sample taken together; therefore, *n* = 862. Body-mass-index (BMI) percentiles were calculated with the R package PAutilities [36] according to CDC (Centers for Disease Control and Prevention) standards. Cohen’s d effect size was calculated for metric scaled, and Cramér’s V for categorial data.

**Table 2:** Would you like support in the interaction with your child during the COVID-19 pandemic?

	**COPSY Sample**			**Waldorf Sample**
**Answer Options**	**1st Wave**	**2nd Wave (*n* = 1625)**	**3rd Wave (*n* = 1618)**	**3rd Wave (*n* = 394)**
Yes	63.0%	58.7% (*n* = 953)	59.9% (*n* = 968)	70.8% (*n* = 279)
…always	-	3.6% (*n* = 58)	3.2% (*n* = 52)	9.9% (*n* = 39)
…rather often	-	9.4% (*n* = 153)	7.0% (*n* = 113)	15.7% (*n* = 62)
…occasionally	-	45.7% (*n* = 742)	49.7% (*n* = 803)	45.2% (*n* = 178)
Never	37.0%	41.3% (*n* = 671)	40.1% (*n* = 650)	29.2% (*n* = 115)

Absolute frequencies and the distribution of the response options: “yes, occasionally”, “yes, rather often”, and “yes, always” were not available for the COPSY sample of the first wave.

**Table 3:** Subgroup analysis based on a median split with respect to living conditions of the Waldorf families.

	Split	Response Rate	Dealing with the Relationships within the Family	Dealing with the Child’s Emotions and Moods	Dealing with the Child’s Behavior	Return of the Child from Isolation	Dealing with the Child’s Academic Demands	Fisher. *p* Value
Access to a garden or terrace	Yes	223/350 (63.7%)	71 (31.8%)	110 (49.3%)	84 (37.7%)	60 (26.9%)	152 (68.2%)	0.954
	No	34/48 (70.8%)	12 (35.3%)	16 (47.1%)	16 (47.1%)	9 (26.5%)	23 (67.7%)	
Number of persons in the household	≥4	195/293 (66.6%)	69 (35.4%)	98 (50.3%)	84 (43.1%)	48 (24.6%)	128 (65.6%)	0.066
	<4	63/109 (57.8%)	14 (22.2%)	28 (44.4%)	16 (25.4%)	22 (34.9%)	47 (74.6%)	
Number of rooms in the house/apartment	≥5	155/243 (63.8%)	51 (32.9%)	74 (47.8%)	57 (36.8%)	42 (27.1%)	102 (65.8%)	0.987
	<5	101/154 (65.6%)	32 (31.7%)	50 (49.5%)	42 (41.6%)	27 (26.7%)	71 (70.3%)	
Number of children aged between 7 and 17 years	>1 child	121/181 (66.9%)	36 (29.8%)	57 (47.1%)	48 (39.7%)	31 (25.6%)	84 (69.4%)	0.944
	1 child	137/220 (62.3%)	47 (34.3%)	69 (50.4%)	52 (37.96%)	39 (28.5%)	91 (66.4%)	

**Table 4:** Mean values ± standard deviations of the global HRQoL score (KIDSCREEN-10 Index, parent-proxy report) for Waldorf and COPSY sample.

	COPSY Sample			Waldorf Sample
	Wave 1	Wave 2	Wave 3	Wave 3
*n*	1586	1625	1618	413
M ± SD	41.70 ± 9.69	41.24 ± 10.33	46.09 ± 11.19	47.33 ± 11.91

## Data Availability

The data presented in this study are available on request from the corresponding author.

## Supplementary Materials

The following supporting information can be downloaded at: https://www.mdpi.com/article/10.3390/ijerph20064756/s1, Table S1: Comparative data of German Waldorf parents (*n* = 3685).
