# Supplementary material for: Lessons Learned—The Impact of the Third Wave of the COVID-19 Pandemic on German Waldorf Parents’ Support Needs and Their Rating of Children’s Health-Related Quality of Life: A Cross-Sectional Online Survey
Source: Int J Environ Res Public Health. 2023 Mar 8;20(6):4756. doi: 10.3390/ijerph20064756 (PMC10049119; doi:10.3390/ijerph20064756)
Supplement: Supplementary file 1 [file ijerph-20-04756-s001.zip › ijerph-2227195-supplementary.pdf]

Table S1. Comparative data of German Waldorf parents (n=3685) [42]

**Demographics**	**Response options**	**Mean / %**
Age	*Father*	48
	Mother	45
Number of children	-	2.39
Marital status	*Single*	4.9%
	Married / in a registered partnership	78.7%
	In a committed relationship	7.8%
	Divorced	7.9%
Education	*Higher education entrance qualification/Abitur/Advanced technical college certificate*	75%
	Waldorf graduation after grade 12	1%
	General certificate of secondary education	20%
	Certificate of secondary education	4%
	No school leaving certificate	0%
Employment status	*Employment*	60%
	Self-employed	27.1%
	Civil service	7.2%
	Worker/employed in agriculture	4%
Occupation	*Full-time employed*	54.6%
	Part-time employed	30.8%
	Housewife, househusband	5.9%
	On parental leave	1.2%
	Unemployed	1.4%
	Retiree/pensioners	1.4%
	Student/in education	1.2%
	Other employment	3.4%
Number of persons in household	-	4.04
